# One-pot assembling pyrroloquinoline quinone glucose dehydrogenase with polydopamine to overcome the reproducibility issues of layer-by-layer electrode development†

**DOI:** 10.1039/d5sd00053j

**Published:** 2025-06-20

**Authors:** Alessandra Cimino, Shixin Wang, Verdiana Marchianò, Angelo Tricase, Angela Stefanachi, Eleonora Macchia, Blanca Cassano, Luisa Torsi, Xiaoming Zhang, Paolo Bollella

**Affiliations:** a Department of Pharmacy-Pharmaceutical Science, University of Bari A. Moro Via E. Orabona 4 70125 Bari Italy; b School of Science, Minzu University of China Beijing 100081 China xmzhang@muc.edu.cn paolo.bollella@uniba.it; c Optoelectronics Research Center, Minzu University of China Beijing 100081 China; d Centre for Colloid and Surface Science, University of Bari A. Moro Via E. Orabona 4 70125 Bari Italy; e Faculty of Science and Engineering, Åbo Akademi University Turku Finland; f Department of Chemistry, University of Bari A. Moro Via E. Orabona 4 70125 Bari Italy

## Abstract

The reproducibility of enzyme-based biosensors remains a critical challenge, particularly in clinical and wearable applications. Here, we present a novel one-pot polydopamine (PDA)-assisted immobilization strategy for pyrroloquinoline quinone-dependent glucose dehydrogenase (PQQ-GDH) on graphite electrodes to address the limitations of conventional layer-by-layer (LbL) methods. The (PQQ-GDH/PDA)_OPA_/G platform demonstrated a uniform and nanostructured enzyme–polymer matrix, confirmed by SEM and spectroscopic characterization, resulting in enhanced surface coverage and enzyme stabilization. Electrochemical analyses revealed an onset potential of +0.19 ± 0.01 V and a maximum current of 0.87 ± 0.08 μA in the presence of glucose. Amperometric calibration yielded a linear range of 0.4–1.2 mM, a sensitivity of 0.47 μA mM^−1^, and a low detection limit of 26 ± 2 μM. Michaelis–Menten kinetic analysis provided an *I*_max_ of 1.13 ± 0.07 μA and a *K*^app^_M_ of 3.11 ± 0.59 mM. Reproducibility was excellent, with relative standard deviations below 8% for all key parameters. The biosensor retained full functionality under physiological conditions (pH 7.2, 37 °C) and exhibited high selectivity against common interferents, including dopamine, uric acid, and ascorbic acid, with signal variations below 5%. Remarkably, the sensor maintained stable responses in artificial serum for over 67 days, confirming its long-term operational stability. These findings highlight the one-pot PDA-based approach as a scalable, reproducible, and biocompatible platform for next-generation glucose biosensors suitable for real-world biomedical monitoring.

## Abstract 2

The reproducibility of enzyme-based biosensors remains a critical challenge, particularly in clinical and wearable applications.

## Graphical abstract

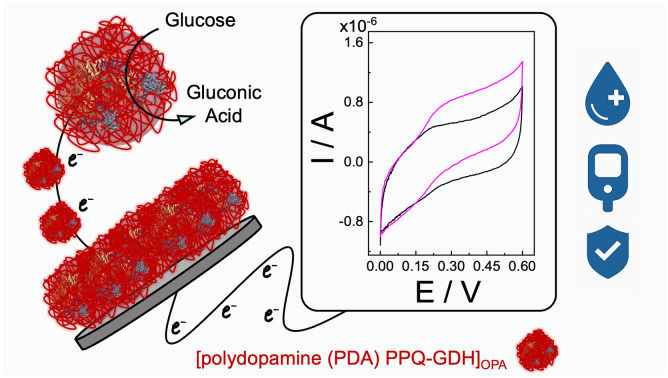

## 1. Introduction

Recently, amperometric enzyme-based biosensors have been rapidly evolving addressing several issues about the electron transfer rate (faster electron transfer can be correlated with the limiting current recorded, hence increasing sensitivity), the amount of enzyme immobilized on the electrode surface (developing a 3D electrode structure to host a high number of enzyme molecules in order to increase sensitivity) and interferences.^1–4^ However, there is a little consideration on the reproducibility and repeatibility of (bio)sensor response, particularly comparing immobilization techniques like layer-by-layer (LbL), covalent linking, self-assembly, physisorption and one-pot assembly.^5–8^

The reproducibility of amperometric enzyme-based biosensors is a crucial factor in ensuring reliable performance, particularly in clinical, environmental, and industrial applications. According to ISO 5725 and ISO 20776-1,^9,10^ reproducibility refers to the degree of agreement between independent measurements conducted under varying conditions, such as different operators, instruments, and laboratories.^11–13^ To meet ISO standards, biosensors must exhibit an intra-assay coefficient of variation (CV) of ≤5% and an inter-assay CV of ≤10%, ensuring consistency within and across multiple tests.^14,15^ Additionally, the standard deviation (SD) should not exceed ±2–3% of the mean signal, while the acceptable accuracy error is generally within ±15%, with a tolerance of up to ±20% for low-concentration analytes.^16,17^ These thresholds help guarantee that amperometric enzyme-based biosensor performance remains stable despite potential variations in enzyme immobilization, electrode composition, and reagent consistency.^18–20^ Compliance with ISO regulations is essential for amperometric enzyme-based biosensor validation, regulatory approval, and practical deployment in diagnostic and monitoring applications.^21^

Enzyme immobilization significantly impacts the reproducibility of amperometric biosensors.^22,23^ The LbL method, despite its versatility, often leads to batch-to-batch variations, non-uniform enzyme distribution, and weak interlayer adhesion, compromising reproducibility and stability.^24–26^ In contrast, one-pot electrode modification provides a more reliable approach by integrating enzyme immobilization and electrode functionalization in a single step.^27–29^ This method ensures homogeneous enzyme distribution, stronger binding interactions, and reduced fabrication variability, enhancing reproducibility and scalability.^30–32^ Thus, one-pot modification represents a superior strategy for developing consistent and high-performance biosensors across various applications.^33^

Polydopamine (PDA)-mediated assembly has shown remarkable potential due to its biocompatibility, adhesive nature, and ability to facilitate electron transfer (ET).^34–36^ PDA, inspired by mussel adhesion mechanisms, is synthesized through the oxidative polymerization of dopamine under alkaline conditions.^37^ Its structure comprises catechol, quinone, and amine groups, which enable strong interactions with biomolecules through mechanisms such as hydrogen bonding, π–π stacking, electrostatic forces, and metal coordination.^38,39^ These interactions are crucial for improving enzyme stability and optimizing electron communication.^40^ In contrast, the conventional LbL deposition technique, which involves the sequential adsorption of oppositely charged layers, often results in random enzyme orientation and low electron transfer efficiency.^41^

Pyrroloquinoline quinone-dependent glucose dehydrogenase (PQQ-GDH) is widely employed in glucose biosensing due to its rapid electron transfer kinetics and stable cofactor structure.^42–44^ The method of immobilization significantly affects biosensor performance parameters such as limit of detection (LoD), sensitivity, dynamic linear range, and storage stability. The self-assembly of PDA with PQQ-GDH is postulated to be driven by multiple cooperative interactions. The catechol and quinone functionalities within PDA facilitate hydrogen bonding and covalent interactions with specific amino acid residues of PQQ-GDH, including lysine, histidine, and cysteine.^45^ Additionally, π–π stacking interactions between PDA's aromatic moieties and the PQQ cofactor contribute to stabilizing the enzyme's conformation, thereby enhancing electron transfer efficiency and catalytic activity.^46^ Electrostatic interactions further play a role in stabilizing the enzyme, as PDA exhibits a zwitterionic nature that can support charge neutrality, leading to improved enzyme retention and activity under physiological conditions.^47^

**Fig. 1:**
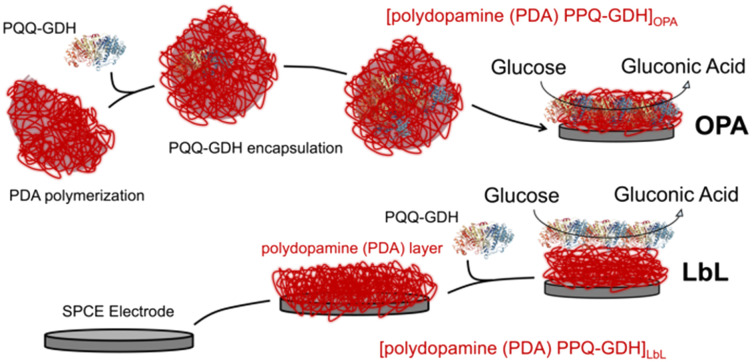
Scheme of the deposition protocol for (PQQ-GDH/PDA)_OPA_/G and (PQQ-GDH/PDA)_LbL_/G electrodes.

In this study, we assess the impact of PDA-one pot assembly (OPA) *versus* LbL deposition on the electrochemical performance of glucose biosensors (Fig. 1). Through techniques such as cyclic voltammetry (CV), electrochemical impedance spectroscopy (EIS), and chronoamperometry (CA), we evaluate key performance metrics including LoD, sensitivity, dynamic linear range, and long-term stability. The results provide insight into the significance of PDA–enzyme interactions towards biosensor performance. We demonstrated that PDA-assisted assembly exhibited better catalytic properties compared to LbL deposition by promoting enzyme stability, optimizing enzyme loading, and facilitating efficient electron transfer pathways. Although polydopamine (PDA)-assisted immobilization strategies have been previously reported, this work introduces a scalable one-pot co-deposition approach with pyrroloquinoline quinone-dependent glucose dehydrogenase (PQQ-GDH), a direct electron transfer enzyme, uniquely demonstrating exceptional reproducibility (RSD <10% for all analytical parameters) and unprecedented operational stability over 67 days—features not addressed in prior PDA-based biosensing studies.

## 2. Experimental

### 2.1 Chemicals and reagents

4-(2-Hydroxyethyl)piperazine-1-ethanesulfonic acid (HEPES), 4-morpholinepropanesulfonic acid (MOPS), tris(hydroxymethyl)aminomethane (Tris), d-glucose, d-galactose, l-lactic acid, potassium chloride (KCl), calcium chloride (CaCl_2_), hydrochloric acid (HCl), potassium hydroxide (KOH), sodium acetate (CH_3_COONa), sodium hydroxide (NaOH), ascorbic acid, d-fructose, dopamine hydrochloride, artificial serum, urea and uric acid were purchased from Merck Millipore (formerly Sigma Aldrich).

Pyrroloquinoline quinone glucose dehydrogenase (PQQ-GDH) was obtained from Toyobo Enzymes. PQQ-GDH (activity 787 U mL^−1^) was dissolved in 10 mM HEPES buffer at pH 7.4 containing 6 mM CaCl_2_ (stored in aliquots at −20 °C).^48^

All solutions were prepared using Milli-Q water (18.2 MΩ cm, Millipore, Bedford, MA, USA). All measurements were conducted under precise temperature regulation using a thermostatic system (±0.01 °C accuracy, LAUDA RM6, Delran, NJ, USA).

### 2.2 Polymerization of dopamine and PQQ-GDH encapsulation

To initiate dopamine (DA) polymerization, a 10 mM tris(hydroxymethyl)aminomethane (Tris) buffer at a pH of 8.6 was prepared.^49^ Dopamine hydrochloride was subsequently added to reach a concentration of 2 mg mL^−1^. The resulting solution was gently stirred at 600 rpm and maintained at 25 °C for 6 hours. As the reaction proceeded, the solution gradually transitioned from colorless to brown, confirming the oxidative self-polymerization of dopamine into polydopamine. To initiate DA polymerization and simultaneously encapsulate PQQ-GDH, a 10 mM Tris buffer was prepared at pH 8.6 (*T* = 25 °C). Dopamine hydrochloride was added to the buffer to achieve a final concentration of 2 mg mL^−1^. Separately, PQQ-GDH was prepared at 787 U mL^−1^ in 10 mM HEPES buffer (pH 7.4) containing 6 mM CaCl_2_, and then combined with the dopamine solution to allow for co-immobilization. The mixed solution was gently stirred at 600 rpm and maintained at 25 °C for 6 hours. This one-step co-deposition approach promotes stable enzyme integration into the PDA network through a combination of hydrogen bonding, π–π interactions, and covalent linkage with PDA quinone/catechol moieties.

### 2.3 Electrode modification

Stencil-printed graphite (SPGE) electrodes (geometric area = 0.1256 cm^2^) were used as working electrodes and printed as previously reported.^50–52^ For the (PQQ-GDH/PDA)_OPA_/G architecture, 5 μL of a freshly prepared mixture of (PQQ-GDH/PDA)_OPA_ was drop-cast on the electrode surface and incubated for 2 hours at 25 °C to enable co-immobilization *via* PQQ-GDH encapsulation within the PDA film. Afterwards, the electrodes were rinsed with HEPES buffer and stored overnight at +4 °C in 10 mM HEPES buffer (pH 7.2) for conditioning and stabilization. For the (PQQ-GDH/PDA)_LbL_/G configuration, electrodes were modified by sequential deposition of 5 μL PDA solution (2 mg mL^−1^ in 10 mM Tris buffer, pH 8.5), incubated for 1 hour to allow film formation, followed by rinsing and drying. Subsequently, 5 μL of the PQQ-GDH solution (the same composition as above) was drop-cast onto the PDA-modified surface and allowed to adsorb for 1 hour at room temperature. The final electrodes were also conditioned overnight at +4 °C in 10 mM HEPES buffer at pH 7.2 prior to use.

### 2.4 Electrochemical, morphological and spectroscopic measurements

Cyclic voltammetry and amperometry experiments were performed using a PalmSens4 electrochemical workstation equipped with PSTrace 5.6v software. All potentials were measured using a BASi Ag|AgCl|3M KCl (all potential values reported in the paper need to be considered toward this reference) and a platinum wire as reference and counter electrodes, respectively. The morphological characterization was conducted using a field emission scanning electron microscope (FE-SEM) model ∑igma Zeiss (Jena, Germany). Images were acquired in top-view using the in-lens detector, with a 5 kV acceleration voltage, 4 mm working distance, 30 μm opening, and no additional sample treatment. ATR-IR analyses were conducted using a Perkin-Elmer Spectrum-Two. The crystal surface was cleaned with 2-propanol before use. After the cleaning solvent evaporation, the background spectrum was acquired against air at a resolution of 1 cm^−1^ over a frequency range of 4000–600 cm^−1^. Substrates were cleaned by sonication in MilliQ water, acetone, and 2-propanol, then modified by drop-casting and incubated at 40 °C for 120 minutes. Spectra were processed using the software provided by the manufacturer (Spectrum10 Std) for initial treatment and Origin2021 for subsequent corrections. Alterations in the secondary structure of PQQ-GDH following immobilization *via* OPA or LbL strategies were examined using circular dichroism (CD) spectroscopy. Spectra were recorded with a J-815 CD spectropolarimeter (JASCO, Easton, MD, USA) by placing the modified ITO electrodes (cat. CEC007, Präzisions Glas & Optik GmbH, Iserlohn, Germany) in a standard 1 cm quartz cuvette containing 10 mM HEPES buffer (pH 7.2). This setup enabled a direct comparison of structural retention between the OPA and LbL immobilization approaches.

## 3. Results and discussion

### 3.1 Electrochemical, spectroscopic and morphological characterization of PQQ-GDH/PDA_LbL_ and PQQ-GDH/PDA_OPA_ immobilization methods

**Fig. 2:**
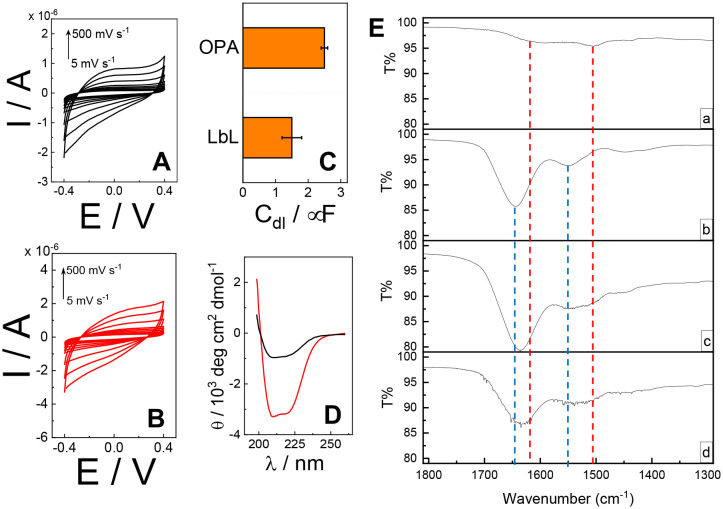
(A) CVs performed for (PQQ-GDH/PDA)_LbL_/G in 10 mM HEPES buffer pH 7.2 + 100 mM KCl in the potential window of −0.4–0.4 V at different scan rates from 5 to 500 mV s^−1^; (B) CVs performed for (PQQ-GDH/PDA)_OPA_/G in 10 mM HEPES buffer pH 7.2 + 100 mM KCl in the potential window of −0.4–0.4 V at different scan rates from 5 to 500 mV s^−1^; (C) Bar diagram reporting *C*_dl_ for (PQQ-GDH/PDA)_OPA_/G and (PQQ-GDH/PDA)_LbL_/G extracted from (A) and (B) through 
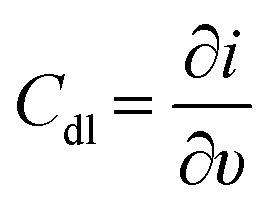
; (D) CD spectra of the (PQQ-GDH/PDA)_LbL_/ITO electrode (black line) and (PQQ-GDH/PDA)_OPA_/ITO electrode (red line) obtained in 10 mM HEPES buffer pH 7.2 + 100 mM KCl; (E) attenuated total reflection-infrared (ATR-IR) spectra recorded for: a) polydopamine, b) PQQ-GDH, c) (PQQ-GDH/PDA)_LbL_/G, and d) (PQQ-GDH/PDA)_OPA_/G.

To investigate the electrode interface properties, cyclic voltammetry (CV) was carried out in 10 mM HEPES buffer pH 7.2 + 100 mM KCl under non-faradaic conditions. Fig. 2A and B illustrate the voltammograms obtained for the (PQQ-GDH/PDA)LbL/G and (PQQ-GDH/PDA)OPA/G electrodes, respectively, across the scan rate range of 5–500 mV s^−1^. Both electrode architectures exhibited capacitive behavior characteristics of surface-confined redox systems, as confirmed by the linear dependence of capacitive current (*I* measured at *E* = 0 V) at different scan rates (*ν*), in accordance with the relation 
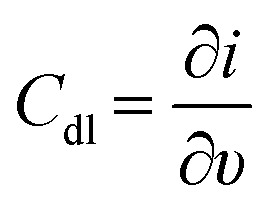
.^53^ Notably, the slope derived from the Δ*I vs. ν* plot was 2.5 ± 0.1 μF for the OPA electrode, indicating a larger electrochemical double-layer capacitance and more effective surface coverage correlated with higher enzyme immobilization density compared to the LbL configuration, reporting 1.5 ± 0.3 μF, as shown in Fig. 2C.

Circular dichroism (CD) spectroscopy was used to evaluate the structural preservation of PQQ-GDH after immobilization. As shown in Fig. 2D, the spectrum for the OPA-based electrode retained the characteristic α-helical signals of the native enzyme, including distinct negative bands near 208 and 222 nm. In contrast, the LbL sample displayed diminished ellipticity and broader spectral features, indicating partial loss of the secondary structure. This suggests that the one-step assembly preserves the enzyme's native conformation more effectively, likely due to a more biocompatible and uniformly distributed PDA–enzyme interface.^54^

In Fig. 2E, the ATR-IR spectra in the range of 1800–1300 cm^−1^ are shown. PDA exhibited two main peaks centered at 1611 ± 1 cm^−1^ and 1504 ± 1 cm^−1^, attributed to the aromatic C–C stretching signals.^55,56^ For PQQ-GDH, three main peaks were observed, corresponding to the characteristic polyamide signals: notably, amide I (1645 ± 1 cm^−1^), amide II (1551 ± 1 cm^−1^), and amide III (1449 ± 1 cm^−1^).^57^ Spectra c and d show a combination of polydopamine and PQQ-GDH signals, with an overlap of the amide I and amide II peaks of PQQ-GDH (blue lines) with the main peaks of polydopamine (red lines).

However, the shape of the amide I signal changes from the pure enzyme layer (spectrum b) to the layer-by-layer samples (spectrum c), and the mixed sample (spectrum d) cannot be explained simply as a combination of the signals from each individual component, especially when comparing the intensity of the peaks in spectra a–d. Therefore, we hypothesize a partial red shift of the amide I peak due to interactions between PDA and polyamide structures, particularly in spectrum d, as also reported by Saurabh *et al.* for similar systems.^58^

Fig. S1† displays the SEM pictures of the electrode surfaces modified *via* the one-pot assembly (OPA, Fig. S1A†) and layer-by-layer (LbL, Fig. S1B†) strategies. The OPA electrode revealed a compact and nanostructured film with a consistent distribution of spherical and granular features with an average diameter of 232 ± 16 nm, suggesting uniform incorporation of the PQQ-GDH enzyme molecules within the polydopamine matrix.^45^ This continuous morphology implies that the co-immobilization of PDA and enzyme molecules during the single-step process leads to a cohesive and homogeneous biofunctional layer. Such uniformity is expected to enhance the effective surface area and promote more efficient electrochemical communication between the enzyme and the electrode interface. In contrast, the LbL-modified electrode exhibits a more irregular surface, characterized by discrete clusters and exposed regions, pointing to a non-uniform assembly. This fragmented structure likely stems from variability in LbL deposition, resulting in inconsistent enzyme distribution and weaker physical integrity of the film.^59^ Hence, SEM characterization confirmed that more organized and densely packed surface architecture results in higher electrochemical double-layer capacitance (*C*_dl_).^60,61^

### 3.2 Characterization of (PQQ-GDH/PDA)_LbL_/G and (PQQ-GDH/PDA)_OPA_/G electrodes under turnover conditions

**Fig. 3:**
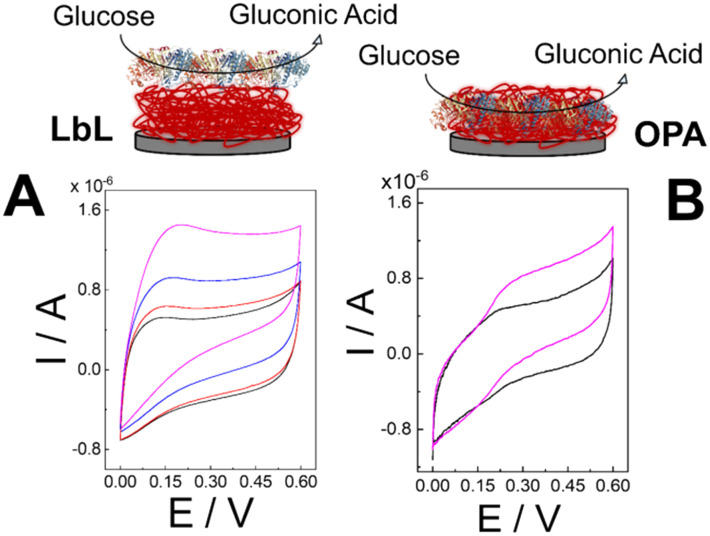
(A) CVs performed for (PQQ-GDH/PDA)_LbL_/G in 10 mM HEPES buffer pH 7.2 + 100 mM KCl at 2 mV s^−1^ containing 0 mM (black curve), 1 mM (red curve), 5 mM (blue curve) and 10 mM (magenta curve) d-glucose (inset: scheme of (PQQ-GDH/PDA)_LbL_/G); (B) CVs performed for (PQQ-GDH/PDA)_OPA_/G in 10 mM HEPES buffer pH 7.2 + 100 mM KCl at 2 mV s^−1^ containing 0 mM (black curve) and 10 mM (magenta curve) d-glucose (inset: scheme of (PQQ-GDH/PDA)_OPA_/G).

The electrocatalytic activity of glucose biosensors was assessed by CV in both the absence and presence of a substrate. Measurements were performed in 10 mM HEPES buffer (pH 7.2) containing 100 mM KCl. For the (PQQ-GDH/PDA)_LbL_/G system, as shown in Fig. 3A, the CV recorded under non-turnover conditions (0 mM glucose, black curve) displayed a capacitive-like CV without redox peaks that could be ascribed to faradaic electron transfer processes between PQQ cofactors and the PDA supporting layer. After the addition of 1 mM d-glucose (red curve), 5 mM d-glucose, and 10 mM d-glucose (magenta curve), an anodic catalytic wave appeared with an onset potential at = 0 V, reaching limiting currents at *E* = +0.35 V of 0.68 ± 0.08 μA, 0.84 ± 0.09 μA and 1.4 ± 0.1 μA, respectively.

Similarly, the (PQQ-GDH/PDA)_OPA_/G electrode (Fig. 3B) in the absence of glucose exhibited a couple of redox peaks (black trace, *I*_pa_ = 0.085 ± 0.009 μA and *I*_pc_ = 0.047 ± 0.007 μA) with a formal redox potential (*E*^0^′) of +0.21 ± 0.01 V, which can be ascribed to the effective enzyme encapsulation within PDA microstructures, enabling an effective electron transfer through the PDA moieties. Upon glucose addition, a catalytic wave exhibited an onset potential (*E*_ONSET_) of +0.19 ± 0.01 V raising up to the limiting current of 0.87 ± 0.08 μA (Fig. 3B, magenta curve). This pronounced CV response reflects more efficient electron mediation, likely due to the uniform co-immobilization of PQQ-GDH and PDA, which facilitates improved electrical communication and substrate accessibility.^45^ The superior performance of the OPA-based electrode highlights the benefits of its nanostructured and cohesive film morphology.

**Fig. 4:**
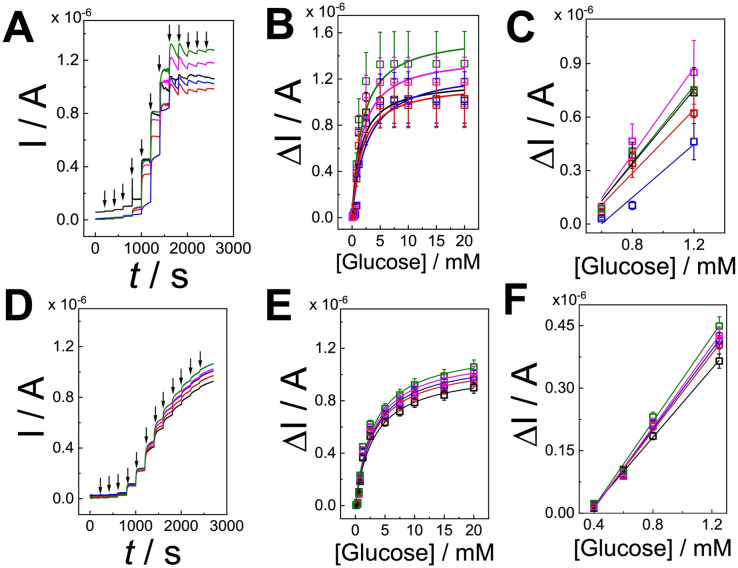
(A) Average amperometric curves performed for (PQQ-GDH/PDA)_LbL_/G in 10 mM HEPES buffer pH 7.2 + 100 mM KCl at *E* = +0.35 V sequentially adding d-glucose from 100 μM to 20 mM (arrows correspond to the addition and different color codes are for all replicates from 1 to 5); (B) calibration curves extracted from amperometric curves performed for (PQQ-GDH/PDA)_LbL_/G in 10 mM HEPES buffer pH 7.2 + 100 mM KCl at *E* = +0.35 V sequentially adding d-glucose from 100 μM to 20 mM (different color codes are for all replicates from 1 to 5); (C) linear dynamic range for (PQQ-GDH/PDA)_LbL_/G; (D) average amperometric curves performed for (PQQ-GDH/PDA)_LbL_/G in 10 mM HEPES buffer pH 7.2 + 100 mM KCl at *E* = +0.35 V sequentially adding d-glucose from 100 μM to 20 mM (arrows correspond to the addition and different color codes are for all replicates from 1 to 5); (E) calibration curves extracted from amperometric curves performed for (PQQ-GDH/PDA)_LbL_/G in 10 mM HEPES buffer pH 7.2 + 100 mM KCl at *E* = +0.35 V sequentially adding d-glucose from 100 μM to 20 mM (different color codes are for all replicates from 1 to 5); (F) dynamic linear range for (PQQ-GDH/PDA)_LbL_/G.

Both (PQQ-GDH/PDA)_LbL_/G and (PQQ-GDH/PDA)_OPA_/G electrodes were evaluated by amperometry upon successive addition of d-glucose in the range of 0.1 to 20 mM (Fig. 4A and D, respectively, black arrows correspond to d-glucose addition). The calibration curve for (PQQ-GDH/PDA)_LbL_/G, reported in Fig. 4B, showed a linear range extending from 0.6 to 1.2 mM (Fig. 4C), with a sensitivity of 0.99 ± 0.2 μA mM^−1^, an average correlation coefficient of 0.998, and a limit of detection (LoD) of 0.4 ± 0.2 mM (RSD = 50.0%, *n* = 50, divided as *n* = 10 on 5 batches). Michaelis–Menten kinetics fitting of the response curve yielded a maximum current (*I*_max_) of 1.2 ± 0.3 μA and an apparent Michaelis–Menten constant (*K*^app^_M_) of 1.9 ± 0.6 mM (Fig. 4B). The obtained *K*^app^_M_ value is comparable with the solution-phase measurements.^42^ This may be ascribed to partial diffusion limitations and non-uniform enzyme orientation across the LbL layers.

The calibration curve for the (PQQ-GDH/PDA)_OPA_/G electrode was recorded in the range of 0.1 to 20 mM (Fig. 4E), with a LoD of 26 ± 2 μM, a linear range extending from 0.4 to 1.2 mM (Fig. 4F), a sensitivity of 0.47 ± 0.02 μA mM^−1^, and a correlation coefficient of 0.99 (RSD = 7.8%, *n* = 50, divided as *n* = 10 on 5 batches). Kinetic analysis revealed an *I*_max_ of 1.13 ± 0.07 μA and a *K*^app^_M_ of 3.1 ± 0.6 mM, which is closer to literature values reported for PQQ-GDH under homogeneous conditions.^42^ These kinetic improvements are likely attributed to the homogenous co-immobilization and enhanced electroactive surface area offered by the OPA strategy, which promotes faster substrate diffusion and more efficient enzymatic turnover.

To evaluate the reproducibility of the two immobilization strategies, key analytical parameters were extracted from multiple calibration experiments and expressed as mean values with associated relative standard deviations (RSD%), as shown in Table 1. The (PQQ-GDH/PDA)_OPA_/G platform demonstrated excellent consistency across all tested metrics, with RSD values well below the 10% acceptance threshold defined for biosensor reproducibility. Specifically, the linear regression coefficient (*R*^2^) for both Michaelis–Menten (MM) and linear fittings remained remarkably stable (RSD ≤ 0.1%), while kinetic parameters such as *I*_max_ (RSD = 5.7%) and *K*^app^_M_ (RSD = 5.1%) exhibited minimal variation across replicates. The sensitivity (slope, RSD = 4.3%) and the limit of detection (LoD, RSD = 7.7%) further confirmed the robust and repeatable behavior of the OPA configuration, suggesting homogeneous enzyme distribution and stable film morphology.

Comparison of analytical and kinetic figures of merit for (PQQ-GDH/PDA)_OPA_/G and (PQQ-GDH/PDA)_LbL_/G

**Table:** 

(PQQ/GDH/PDA)_OPA_/G							
	BATCH #1 *n* = 10	BATCH #2 *n* = 10	BATCH #3 *n* = 10	BATCH #4 *n* = 10	BATCH #5 *n* = 10	Average	RSD %
*R* ^2^ MM fitting	0.978	0.976	0.976	0.975	0.976	0.976	**0.1**
*I*_max_/μA	1.04 ± 0.06	1.11 ± 0.08	1.13 ± 0.07	1.17 ± 0.06	1.21 ± 0.05	1.13 ± 0.06	**5.7**
*K*_M_/mM	3.2 ± 0.2	3.1 ± 0.1	3.1 ± 0.2	3.2 ± 0.1	3.1 ± 0.2	3.1 ± 0.2	**5.1**
*R* ^2^ linear fitting	0.999	0.997	0.998	0.998	0.998	0.998	**0.1**
Slope/μA mM^−1^	0.41 ± 0.02	0.46 ± 0.01	0.47 ± 0.03	0.49 ± 0.03	0.51 0.02	0.47 ± 0.02	**4.3**
LoD/μM	27 ± 2	28 ± 2	26 ± 1	25 ± 3	24 ± 2	26 ± 2	**7.7**
DLR	0.4–1.2

**Table:** 

(PQQ/GDH/PDA)_LbL_/G							
	BATCH #1 *n* = 10	BATCH #2 *n* = 10	BATCH #3 *n* = 10	BATCH #4 *n* = 10	BATCH #5 *n* = 10	Average	RSD %
R^2^ MM fitting	0.915	0.915	0.913	0.906	0.915	0.91	**0.5**
*I*_max_/μA	1.1 ± 0.2	1.4 ± 0.3	1.3 ± 0.2	1.2 ± 0.4	1.2 ± 0.2	1.2 ± 0.3	**21.0**
*K*_M_/mM	1.8 ± 0.5	1.9 ± 0.6	2.7 ± 0.9	1.6 ± 0.5	1.7 ± 0.6	1.9 ± 0.6	**32.0**
*R* ^2^ linear fitting	0.985	0.985	0.991	0.985	0.985	0.99	**0.2**
Slope/μA mM^−1^	1.22 ± 0.22	1.08 ± 0.19	1.04 ± 0.14	0.89 ± 0.16	0.74 ± 0.13	0.99 ± 0.17	**17.0**
LoD/mM	0.4 ± 0.1	0.3 ± 0.1	0.3 ± 0.2	0.4 ± 0.2	0.5 ± 0.2	0.4 ± 0.2	**50.0**
DLR	0.6–1.2						

Conversely, the (PQQ-GDH/PDA)_LbL_/G platform displayed significantly higher variability, with several parameters exceeding the 10% threshold. The *I*_max_ (RSD = 21%), *K*^app^_M_ (RSD = 32%), and slope (RSD = 17%) all indicated substantial inter-electrode variation, likely arising from batch-to-batch inconsistencies and non-uniform enzyme layering inherent to the layer-by-layer assembly process. Moreover, the standard deviations from curve fitting in both MM and linear models reached up to 21%, further highlighting the limited reproducibility of the LbL approach. While the linear correlation coefficients (*R*^2^ ≈ 0.99) remained acceptable, the broader variation in sensitivity and kinetic performance undermines the robustness of this configuration for analytical deployment. These results clearly demonstrate that the one-pot assembly (OPA) strategy yields superior reproducibility, meeting international standards for biosensor precision and supporting its suitability for clinical and wearable applications.

### 3.3 pH, temperature, selectivity and operational stability of the (PQQ-GDH/PDA)_OPA_/G electrode in artificial serum

**Fig. 5:**
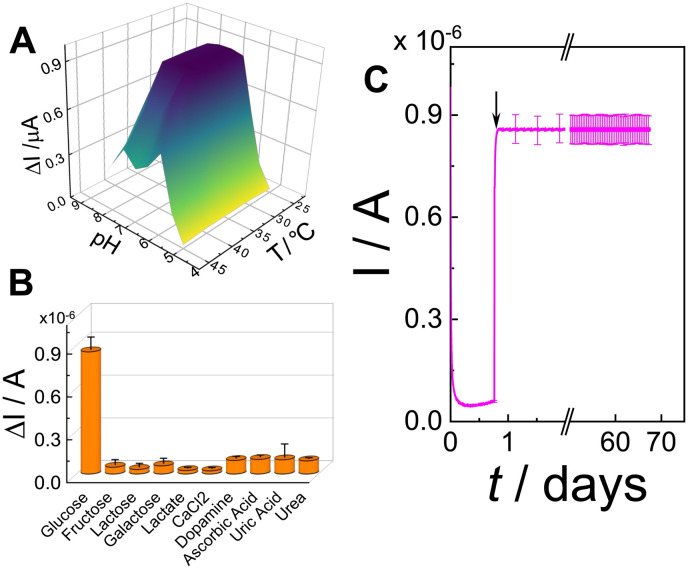
(A) pH and temperature effects on current responses obtained from amperometric curves performed for (PQQ-GDH/PDA)_OPA_/G in 10 mM HEPES buffer pH 7.2 + 100 mM KCl at *E* = +0.35 V adding 10 mM d-glucose; (B) effects of interferents on d-glucose detection with (PQQ-GDH/PDA)_OPA_/G: d-glucose (10 mM), d-fructose (1 mM), β-lactose (1 mM), d-galactose (1 mM), l-lactate (1 mM), CaCl_2_ (1 mM), dopamine (1 nM), ascorbic acid (100 μM), uric acid (400 μM), and urea (7.5 mM); (C) continuous amperometric response for (PQQ-GDH/PDA)_OPA_/G in artificial serum at *E* = +0.35 V obtained by adding 10 mM d-glucose (the arrow corresponds to the addition).

The influence of pH and temperature on the current response was investigated by amperometry in 10 mM HEPES buffer (at different pH values and temperature values) + 100 mM KCl containing 10 mM d-glucose (Fig. 5A). The sensor exhibited maximum response under physiological conditions (pH 7.2, 37 °C), consistent with the native catalytic environment of PQQ-GDH. Outside this optimal range, a progressive decline in signal was recorded, likely due to conformational changes in the enzyme or compromised cofactor interactions, ultimately reducing the bioelectrocatalytic activity.^3^

The selectivity of the proposed biosensor platform was further examined by comparing the amperometric response to glucose with those to several potential interferents commonly present in blood plasma, including d-fructose (1 mM), β-lactose (1 mM), d-galactose (1 mM), l-lactate (1 mM), CaCl_2_ (1 mM), dopamine (1 nM), ascorbic acid (100 μM), uric acid (400 μM), and urea (7.5 mM), each tested at their physiological levels (Fig. 5B). All interferents produced negligible current responses relative to glucose, with signal variations remaining below 5% of the glucose response. Such high selectivity can be attributed to the substrate-selective nature of PQQ-GDH and the compact PDA–enzyme network, which may sterically hinder access to nonspecific redox-active species or restrict their diffusion to the electrode surface.

The (PQQ-GDH/PDA)_OPA_/G electrode was also validated by measuring glucose in artificial serum under flow-injection conditions (Fig. 5C). Upon the addition of 10 mM d-glucose, a sharp and stable current response was observed with no significant baseline drift or loss in signal intensity over 67 days, confirming the anti-fouling characteristics of the OPA configuration. The same experiment was also performed in artificial human serum (red curve) and artificial human sweat (Fig. S2†) retaining 95% and 82%, respectively. These results suggest the platform's strong potential for deployment in biologically relevant fluids for diagnostic and wearable applications.

Table S1† presents a comparative overview of the key analytical figures of merit for the (PQQ-GDH/PDA)_OPA_/G electrode and other glucose biosensing platforms reported in the recent literature.^43,62–67^ The (PQQ-GDH/PDA)_OPA_/G configuration operates at an applied potential of +0.35 V *vs.* Ag/AgCl, with a limit of detection (LoD) of 26 μM and a linear dynamic range (LDR) spanning from 0.4 to 1.2 mM. Importantly, this system demonstrates exceptional operational stability, retaining 95% of its initial response after 67 days of continuous measurement, highlighting its potential for long-term deployment in real-world applications.

Compared to other enzyme–electrode assemblies, several platforms report lower LoDs (*i.e.*, PQQ-GDH/PTh/MWCNT/Au (1 μM) and PQQ-GDH/PAN-PABSA/ITO (2.5 μM)). However, these systems are limited in stability to 15 and 30 days, respectively. The PQQ-GDH@SWCNT-APPA-1.15 electrode achieves a low LoD of 5 μM within a narrow linear range (0.01–0.1 mM), but exhibits acceptable performance over only 24 hours. Platforms based on mediator systems such as Ru-GDH/PDA-MWCNT/SPCE or FAD-GDH/DCPIP@PDA-MWCNT/GCE also show wide linear ranges, but lack extended stability data. Overall, (PQQ-GDH/PDA)_OPA_/G offers a competitive balance between sensitivity, detection range, and long-term stability, supporting its applicability for continuous glucose monitoring in both clinical and wearable biosensing technologies.

## Conclusions

In this work, we demonstrated the effectiveness of a one-pot polydopamine (PDA)-assisted assembly strategy for the immobilization of pyrroloquinoline quinone-dependent glucose dehydrogenase (PQQ-GDH) onto glassy carbon electrodes, providing a robust and reproducible glucose biosensing platform. Compared to the conventional layer-by-layer (LbL) approach, the (PQQ-GDH/PDA)_OPA_/G configuration exhibited superior analytical performance, particularly in terms of reproducibility, sensitivity, and operational stability. The one-pot assembly yielded a uniform, nanostructured enzyme–PDA composite, as confirmed by SEM, with significantly enhanced electrochemical double-layer capacitance (2.5 ± 0.1 μF) and preserved enzyme conformation, as supported by circular dichroism and ATR-IR spectroscopy.

Electrochemical analyses under turnover conditions revealed a well-defined electrocatalytic wave with an onset potential of +0.19 ± 0.01 V and a limiting current of 0.87 ± 0.08 μA upon glucose addition. Amperometric calibration showed a linear dynamic range between 0.4 and 1.2 mM, a sensitivity of 0.47 ± 0.02 μA mM^−1^, and a low detection limit of 26 ± 2 μM. Michaelis–Menten kinetic fitting provided an apparent *K*_M_ of 3.1 ± 0.2 mM and a maximal current (*I*_max_) of 1.13 ± 0.06 μA, closely matching literature-reported values for the enzyme in solution. Reproducibility metrics met the ISO and ICH acceptance criteria, with all key analytical parameters showing relative standard deviations (RSDs) below 8%—including RSDs of 5.1% for *K*_M_, 5.7% for *I*_max_, and 4.3% for sensitivity.

The platform retained high operational stability under physiological conditions (pH 7.2, 37 °C), excellent anti-fouling properties in artificial serum, and strong selectivity against physiological interferents such as uric acid, ascorbic acid, dopamine, and urea, with signal interference below 5%. The (PQQ-GDH/PDA)_OPA_/G electrode demonstrated excellent long-term stability in artificial serum under flow-injection conditions, maintaining a stable and drift-free current response upon 10 mM d-glucose addition for over 67 days, highlighting its strong anti-fouling properties and suitability for prolonged operation in complex biological media. These results confirm the suitability of the one-pot PDA-based immobilization strategy for developing reliable, scalable, and biocompatible glucose biosensors, particularly for applications in wearable and clinical diagnostics where high reproducibility and robustness are essential. These findings validate the one-pot PDA-based strategy as a reliable and scalable approach for glucose biosensors, well-suited for wearable and clinical applications requiring high reproducibility and stability.

## Author contributions

A. C., S. W. and B. C. performed the measurements and data analysis. A. T., S. W., and V. M. wrote part of the manuscript. E. M., A. S., X. Z., P. B. and L. T. supervised A. T. and S. W. on data collecting and analysis. L. T., E. M., X. Z. and P. B. conceived the experimental work, revised the manuscript and are responsible for funding acquisition. The final version was approved by all authors.

## Conflicts of interest

There are no conflicts to declare.

## Supplementary Material

SD-004-D5SD00053J-s001

## Data Availability

Data for this article are available at Zenodo at https://doi.org/10.5281/zenodo.15172113.
