# Supplementary material for: One-pot assembling pyrroloquinoline quinone glucose dehydrogenase with polydopamine to overcome the reproducibility issues of layer-by-layer electrode development
Source: Sens Diagn. 2025 Jun 20;4(9):750–8. doi: 10.1039/d5sd00053j (PMC12231961; doi:10.1039/d5sd00053j)
Supplement: SD-004-D5SD00053J-s001 [file SD-004-D5SD00053J-s001.pdf]

Supplementary Information (SI) for Sensors & Diagnostics.
This journal is © The Royal Society of Chemistry 2025

Electronic Supporting Information

One-pot Assembling Pyrroloquinoline Quinone Glucose Dehydrogenase with Polydopamine to Overcome the Reproducibility Issues of Layer-by-Layer Electrode Development

Alessandra Cimino[1], Shixin Wang[2,3], Verdiana Marchianò[1,4], Angelo Tricase[1,4], Angela Stefanachi[1], Eleonora Macchia[1,4,5], Luisa Torsi[4,6], Xiaoming Zhang[2,3,*], Paolo Bollella[2,4,6,*]

[1] Department of Pharmacy-Pharmaceutical Science, University of Bari A. Moro, Via E. Orabona 4, 70125 Bari, Italy
[2] School of Science, Minzu University of China, Beijing 100081, China
[3] Optoelectronics Research Center, Minzu University of China, Beijing 100081, China
[4] Centre for Colloid and Surface Science, University of Bari A. Moro, Via E. Orabona 4, 70125 Bari, Italy
[5] Faculty of Science and Engineering, Åbo Akademi University, Turku, Finland
[6] Department of Chemistry, University of Bari A. Moro, Via E. Orabona 4, 70125 Bari, Italy

Corresponding authors: Prof. Xiaoming Zhang and Dr. Paolo Bollella email: xmzhang@muc.edu.cn and paolo.bollella@uniba.it

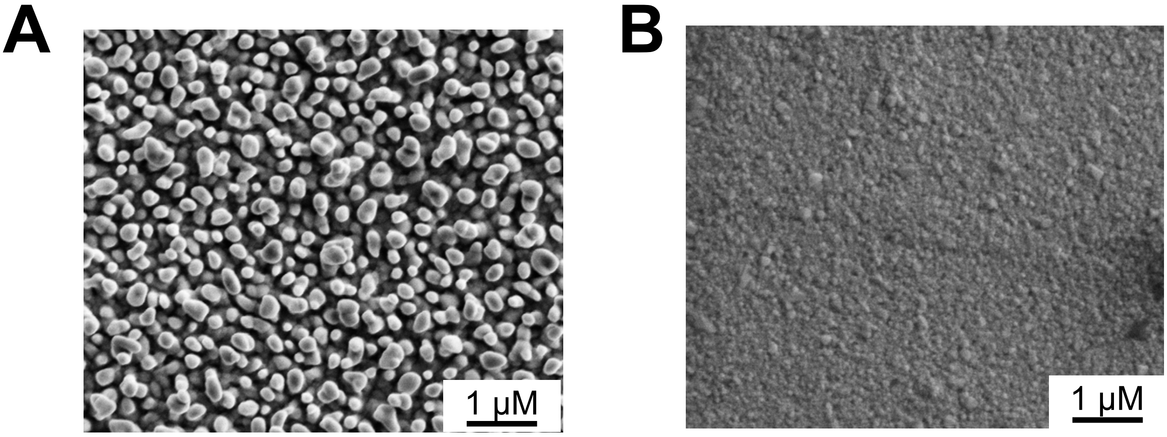

Fig. S1. SEM pictures for $(PQQ\text{-}GDH/PDA)_{OPA}/G$ electrode **(A)** and $(PQQ\text{-}GDH/PDA)_{LbL}/G$ electrode **(B)**.

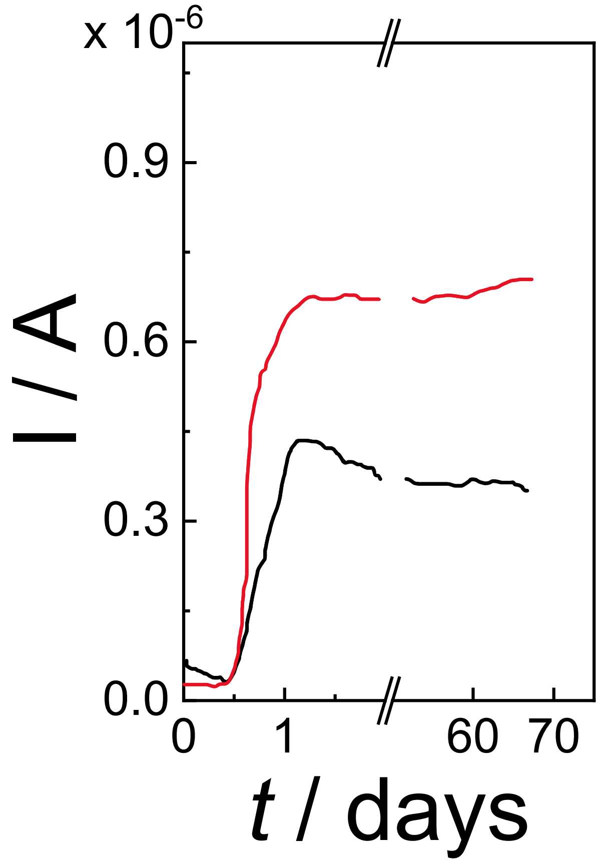

Fig. S2 Continuous amperometric response for (PQQ-GDH/PDA)OPA/G in artificial human blood (red curve) and artificial human sweat (black curve) at E= +0.35 V obtained adding 10 mM D-glucose

Electrode Platform	E_{appl} / V	LoD / µM	DLR / mM	Operational Stability /days	Ref.
**Ru-GDH/PDA-MWCNT/SPCE**	+0.3	94	0.1-30	n.a.	[1]
**FAD-GDH/DCPIP@PDA-MWCNT/GCE**	+0.05 (0)*	n.a.	0-10 (0-20)*	3 days	[2]
**GDH-BSA/GDH/NAD^+/GONs/GCE**	+0.2	n.a.	0-20	n.a.	[3]
**PQQ-GDH@SWCNT-APPA-1.15**	+0.35	5	0.01-0.1	89% of initial signal retained after 24 hour	[4]
**PQQ-GDH/p(MG)-NPs/GPE**	0	10	up to 1	n.a.	[5]
**PQQ-GDH/PTh/MWCNT/Au**	0	1	0.05-0.5	70% signal retained after 15 days	[6]
**PQQ-GDH/PAN-PABSA/ITO**	+0.35	2.5	0.0025-1	65% of initial signal retained after 30 days	[7]
**$(PQQ\text{-}GDH/PDA)_{OPA}/G$**	+0.35	26	0.4-1.2	95% of signal retained after 67 days of continuous operation	This work

Tab S1. Comparison of key analytical figures of merit: potential applied (E_{appl}, V), limit of detection (LoD), dynamic linear range (DLR) and operational/storage stability. Abbreviations: (APPA-1.5) 4-Aminophenylphosphonic Acid, (BSA) Bovine Serum Albumin, (DCNQ) 7,7,8,8-Tetracyanoquinodimethane Derivative (alternative synthetic mediator)*, (DCPIP) 2,6-Dichlorophenolindophenol (synthetic electron mediator), (FAD-GDH) Flavin Adenine Dinucleotide–Dependent Glucose Dehydrogenase, (GCE) Glassy Carbon Electrode, (GDH) Glucose Dehydrogenase (cofactor unspecified), (GONs) Graphene Oxide Nanosheets, (GPE) Graphite Paste Electrode, (ITO) Indium Tin Oxide, (MWCNT) Multi-Walled Carbon Nanotubes, (NAD^+) Nicotinamide Adenine Dinucleotide (oxidized form), (p(MG) Poly(methylene green), (PABSA) Poly(3-Aminobenzoic Acid-co-3-Aminobenzenesulfonic Acid), (PAN) Polyacrylonitrile, (PDA) Polydopamine, (PQQ-GDH) Pyrroloquinoline Quinone–Dependent Glucose Dehydrogenase, (PTh) Polythiophene, (Ru) Ruthenium-based redox mediator ($Ru(bpy)_2$), (SPCE) Screen-Printed Carbon Electrode, (SWCNT) Single-Walled Carbon Nanotubes.

Notes and References

1 W.-Y. Jeon, H.-H. Kim and Y.-B. Choi, *Membranes*, 2021, **11**, 384.
2 R. Cohen, Y. Cohen, D. Mukha and O. Yehezkeli, *Electrochimica Acta*, 2021, **367**, 137477.
3 N. Maleki, S. Kashanian, M. Nazari and N. Shahabadi, *IEEE Sens. J.*, 2019, **19**, 11988–11994.
4 A. F. Quintero-Jaime, F. Conzuelo, D. Cazorla-Amorós and E. Morallón, *Talanta*, 2021, **232**, 122386.
5 M. A. Komkova, A. S. Alexandrovich and A. A. Karyakin, *Talanta*, 2024, **267**, 125219.
6 G. Fusco, G. Göbel, R. Zanoni, M. P. Bracciale, G. Favero, F. Mazzei and F. Lisdat, *Biosens. Bioelectron.*, 2018, **112**, 8–17.
7 J. Gladisch, D. Sarauli, D. Schäfer, B. Dietzel, B. Schulz and F. Lisdat, *Sci. Rep.*, 2016, **6**, 19858.
